# Preparation and Characterization of Dyed Corn Straw by Acid Red GR and Active Brilliant X-3B Dyes

**DOI:** 10.3390/ma12213483

**Published:** 2019-10-24

**Authors:** Yanchen Li, Beibei Wang, Yingni Yang, Yi Liu, Hongwu Guo

**Affiliations:** 1 Key Laboratory of Wooden Material Science and Application, College of Material science And Technology, Beijing Forestry University, Beijing 100083, China; lyc100083@163.com (Y.L.); SyliaWong@163.com (B.W.); yyingni_0922@163.com (Y.Y.); 2 Key Laboratory of Wood Science and Engineering, College of Material science And Technology, Beijing Forestry University, Beijing 100083, China

**Keywords:** corn straw, pretreatment, dyeing, chemical structure

## Abstract

Corn straw is a kind of biomass material with huge reserves, which can be used in plate processing, handicraft manufacturing, indoor decoration, and other fields. To investigate the dyeing mechanism of corn straw with different dyes, corn straw was pretreated and dyed with Acid Red GR and Brilliant Red X-3B. The dyeing properties and light resistance of the two dyes were analyzed by dyeing rate, photochromaticity, FTIR, SEM, and water-washing firmness. The results showed that the structure and stability of the dyes were the main factors which influenced fading. A bleaching pretreatment could remove the waxiness of the corn straw epidermis and increase the porosity on the surface of the straw, which accelerated the photochromic coloring of the corn straw skin. The corn straw dyed with both dyes had good light resistance, but the straw dyed with Reactive Brilliant Red X-3B had higher dyeing rate, brighter color, and higher photochromaticity than the straw dyed with Acid Red GR. FTIR and water-washing firmness showed that Acid Red GR mainly bound to lignin, while Reactive Brilliant Red X-3B mainly bound to cellulose, hemicellulose, and lignin in corn straw through covalent bonds, which increased the coloring rate.

## 1. Introduction

With changes in the quantity and structure of the global forest, the available timber resources for human production have been significantly reduced, and crop straws with a fast green growth cycle have gradually received attention [1,2,3,4]. Corn straw is a natural and renewable biomass resource, mainly composed of cellulose, hemicellulose, and lignin [5]. It has excellent physical and mechanical properties and can even be used as a substitute for wood through some innovative technologies for furniture, interior decoration, and artistic creation. The color of straw is an important indicator of its value [6,7]. However, the waxy layer on the surface of corn straw makes it difficult to be dyed. There is not enough research on how dye molecules bind to corn straw. Furthermore, dyed straw is an excellent light absorber due to the presence of chromophores as phenolic hydroxyl groups, aromatic skeleton, double bonds, and carbonyl groups in the lignin and extractives molecules it contains. These colored unsaturated compounds may cause changes in the surface color and produce new aromatic and other chromophores after irradiation [8,9,10,11,12]. The dyes are also predisposed to fade through either oxidation or reduction reactions when stimulated by light radiation [13,14]. 

At present, research of dyed corn straw and photochromism is not systematic. In order to compare how different dyes bind to corn straw and how the surface color changes after irradiation, this study drew lessons from the theory and process of wood dyeing to dye corn straw, selecting acid dyes and reactive dyes which are commonly used for wood dyeing. Acid Red GR and Active Brilliant Red X-3B were selected as dyeing agents to systematically explore the dyeing of corn straw after pretreatment. Dyeing rate, photochromism, FTIR, SEM, and water-washing firmness were used to analyze the dyeing, light resistance, chemical structure, microstructure, and water-washing firmness of corn straw. This study has a great practical significance to optimize the decorative color of corn straw and obtain a rich color system.

## 2. Materials and Methods

### 2.1. Materials

**Figure 1:**
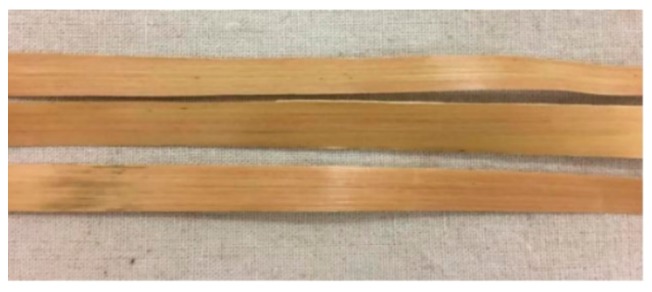
Corn straw epidermis.

Corn straw epidermis: the straw was selected in Fengning, Hebei, as shown in Figure 1. Corn straw with uniform texture and no defect on the surface was cut into 50 mm × 10 mm × 0.1 mm pieces, which were put in black plastic bags for later utilization.

**Figure 2:**
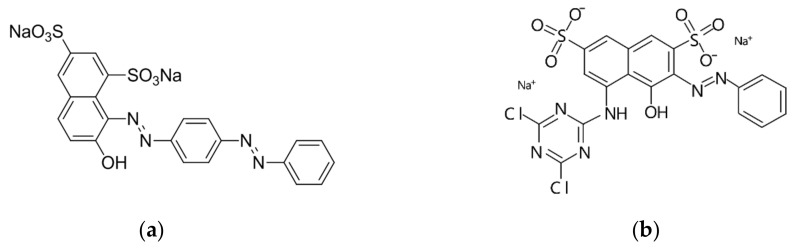
Molecular structures of Acid Red GR (**a**) and Reactive Brilliant Red X-3B (**b**).

Dyes: Acid Red GR and Reactive Brilliant Red X-3B were provided by the Second Plant Dye Chemical Company (Tianjin, China). Their molecular structure formulas are shown in Figure 2.

Reagents: sodium chloride (NaCl), 30% dilute sulfuric acid (H_2_SO_4_), soda ash (Na_2_CO_3_), sodium hydroxide (NaOH), hydrogen peroxide (H_2_O_2_).

Instruments: constant-temperature water bath (HH-4, Kai hang, Shanghai, China); UV–visible spectrophotometer (Genesys10 UV-335903, Thermo Scientific Spectronic, Waltham, MA, USA); automatic spectrophotometer (MF-FS97Pro, Med Future, Shandong, China); solar weather tester (MQ-UV-2, ZKMQ, Tianjin, China); Fourier infrared spectrometer (Nicolet Nexus 670, Waltham, MA, USA); colorimeter (WR10, FRU, China); fourier transform infrared spectrometry(Avatar-380, Thermo-Nicolet, Waltham, MA, USA); scanning electron microscope (Sigma500, ZEISS, Jena, Germany)

### 2.2. Methods

#### 2.2.1. Pretreatment

The specimens were immersed in H_2_O_2_ with a solute mass fraction of 27%, avoiding superposition between the specimens. The solution was heated to 50 °C, and the samples were taken out after 30 min. The surface residue was washed with deionized water at room temperature. After air-drying, the specimens were immersed in NaOH solution with solute mass fraction of 0.1% at room temperature, avoiding superposition among the specimens. After 30 min of treatment, the specimens were air-dried to a moisture content of 8%.

#### 2.2.2. Dyeing Method

Acid Red GR is a cationic dye existing in the form of sodium sulfonate in water, while Reactive Brilliant Red X-3B exists in the form of an anion. The cellulose of corn straw is also charged in aqueous solution as an anion, so there is a repulsive force between Reactive Brilliant Red X-3B and straw cellulose, resulting in low dye utilization. By adding an inorganic salt, NaCl, a large amount of Na^+^ can promote the activity of the dye molecules in water to achieve the purpose of dyeing. Furthermore, NaCl is also good at increasing the coloring rate of the dye molecules. In order to ensure accurate test results, repeated each dyeing test three times.

(1) Acid Red GR staining

Step 1: An atmospheric-pressure dip-dye method was used for wood dyeing. A 0.5% Acid Red GR solution in distilled water (w/v) of at 25 °C was prepared. The pH value of the solution was adjusted to 4.0 by addition of 10% H_2_SO_4_ (w/w). Gaps were left between the samples to avoid overlapping and to ensure dye homogeneity.
Step 2: A bath ratio of 1:20 (V_straw_/V_dye solution_) was used, and the straw was placed into an 50 °C electrically heated thermostatic water bath dye vat for 1 h. After 15 min, a cup of 1% NaCl solution was added to assist the dyeing process. After 30 min, another cup of 1% NaCl solution was added. After dyeing, all dyed straw was washed with distilled water and then air-dried to a moisture content of 12%.

(2) Reactive Brilliant Red X-3B staining

Step 1: Refer to Acid Red GR staining, but the pH value of the solution was adjusted to 10 by addition of 10% Na_2_CO_3_ (w/w).
Step 2: Refer to Acid Red GR staining, but after 30 min, another cup of 1% NaCl and 1% Na_2_CO_3_ solution was added.

**Figure 3:**
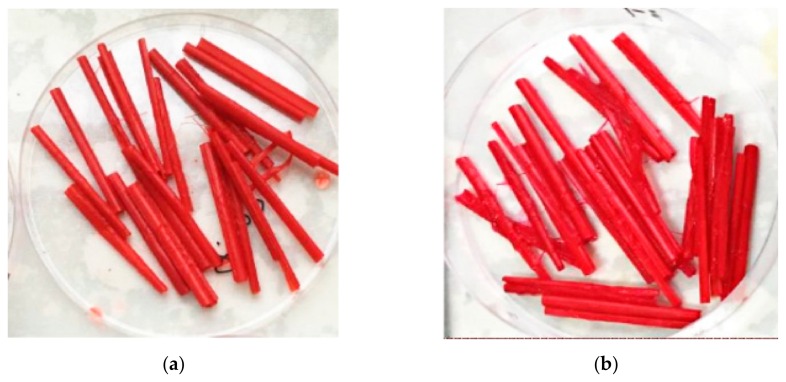
Dyeing effects Acid Red GR (**a**) and Reactive Brilliant Red X-3B on corn straw (**b**).

The dyeing effects of Acid Red GR and Reactive Brilliant Red X-3B on corn straw are shown in Figure 3.

#### 2.2.3. Dyeing Rate Calculation

The maximum absorption spectrum curves of Acid Red GR and Reactive Brilliant Red X-3B were measured by a ultraviolet spectrophotometer, and the absorbance of the dye solution before and after dyeing was measured at the maximum absorption wavelength. The dye uptake rate C_t_ was calculated as follows:

C_t_ (%) = (A_0_ − A_1_) × 100%/A_0_ (1)

In the formula, C_t_ is the dye uptake, A_0_ is the absorbance before dyeing, A_1_ is the absorbance after dyeing.

#### 2.2.4. Chromaticity Value Calculation

The 1976 International Illumination Commission CIE (L*a*b*) standard colorimetric characterization system is used for color measurement in the case of small chromatic aberrations. By comparing the chromaticity values of Acid Red GR and Reactive Brilliant Red X-3B, the light resistance level of the dyed straw could be analyzed and calculated as follows:

ΔE* = [(ΔL*)^2^ + (Δa*)^2^ + (Δb*)^2^]^1/2^ (2)

where ΔE* indicates the degree of total color change, L* stands for lightness from 0 (for black) to 100 (for white), a* represents the red-green chromaticity coordinates (+a* is for red, −a* for green), b* denotes the yellow-blue chromaticity index (+b* is for yellow,−b* for blue), ΔL*, Δa*, and Δb* are the differences of the values of L*, a*, and b* before and after treatment, respectively. The unit of chromaticity value is represented by “NBS”. When the value of ΔE* is 1NBS, it is equivalent about 5 times to the visual recognition threshold. There is approximately such a correspondence between color difference and visual perception: 0.0~0.50, the feel of visual perception is gentle; 0.5~1.50, the feel of visual perception is slight; 1.50~3.0, the feel of visual perception is noticeable; 3.0~6.0, the feel of visual perception is appreciable; 6.0 or more, the feel of visual perception is violent.

The test samples were prepared by laminating the straw which was dyed by Acid Red GR and Reactive Brilliant Red X-3B. Each test piece was placed in a colorimeter (WR10, FRU, China) using a D65 standard illuminant and 10° standard observer for 50 h ultraviolet-lamp irradiation test. The chromatic value ΔE* of the samples were measured after irradiation at each wavelengths of light source for 0, 1, 2, 5, 10, 20, 30, and 50 h, respectively. 

#### 2.2.5. Surface Chemical Structure Analysis

The chemical composition and structure of corn stalks are complex. During the dyeing process, the dye molecules have a large volume, which makes it difficult for them to pass through the microporous structure of the straw, so they are easily adsorbed by the straw components. Therefore, the presence of dyes in straw can be utilized. Attenuated total reflectance–Fourier-transform infrared spectrometry (ATR–FTIR) was performed for the characterization of the surface chemical structure of the dyed straw. All samples were placed on the diamond crystal of the ATR–FTIR spectrometer (Avatar-380, Thermo-Nicolet, Waltham, MA, USA), and the spectra were collected in transmittance mode by 64 scans in the range of 4000–400 cm^−1^ at a resolution of 8 cm^−1^.

#### 2.2.6. Microstructure Analysis

The untreated corn straw, the straw treated by the pretreatment process, and the straw dyed with the two dyes were cut out transversely to make slices, and after gold plating by an ion-coating machine, the images were observed by SEM (Sigma 500, ZEISS, Jena, Germany).

#### 2.2.7. Water-Washing Firmness

Straw dyed by Acid Red GR and Reactive Brilliant Red X-3B was made into straw panels, and the surface chromaticity value ΔE* was measured by an automatic spectrophotometer. Then, the straw was placed in a 1000 ml beaker, and the beaker was placed in an electrically heated thermostatic water bath and soaked at 65 °C for 2 h. After soaking, the straw was taken out and air-dried. Then, the surface chromaticity value ΔE* was measured. 

## 3. Results

### 3.1. Dyeing Rate

The average dyeing rates of Acid Red GR and Reactive Brilliant Red X-3B were calculated in three tests, as shown in Table 1. 

The dyeing rate of Reactive Brilliant Red X-3B was 46%, and that of Acid Red GR was 29%. The dyeing rate of Reactive Brilliant Red X-3B was much higher than that of Acid Red GR, and both dyes all had good dyeing ability acting on corn straw. According to studies on wood dyeing with acid dyes and reactive dyes, acid dyes mainly act on lignin, and the binding mode of dyes to corn stalks is physical adsorption [15,16]. However, the epidermis of corn straw contains a very high amount of cellulose, so the combination of acid dye and straw is limited. Reactive dyes mainly act on cellulose and hemicellulose and react with them to form covalently bound reactive groups [17,18]. When NaCl was added to the reactive dye solution, the negative-charge repulsion between the straw fiber surface and the dye anions in the solution was weakened, greatly improving the opportunity for dye molecules to approach the straw fiber. However, when NaCl was added into the acid dye solution, it only improved the fastness of dye molecules adsorption on the straw epidermis but did not promote the covalent binding between dye molecules and straw components. Therefore, Reactive Brilliant Red X-3B had higher dyeing rate of corn straw epidermis.

### 3.2. Chromatic Value

**Figure 4:**
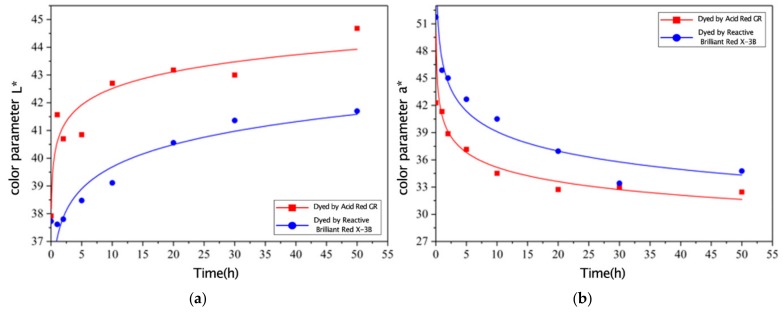

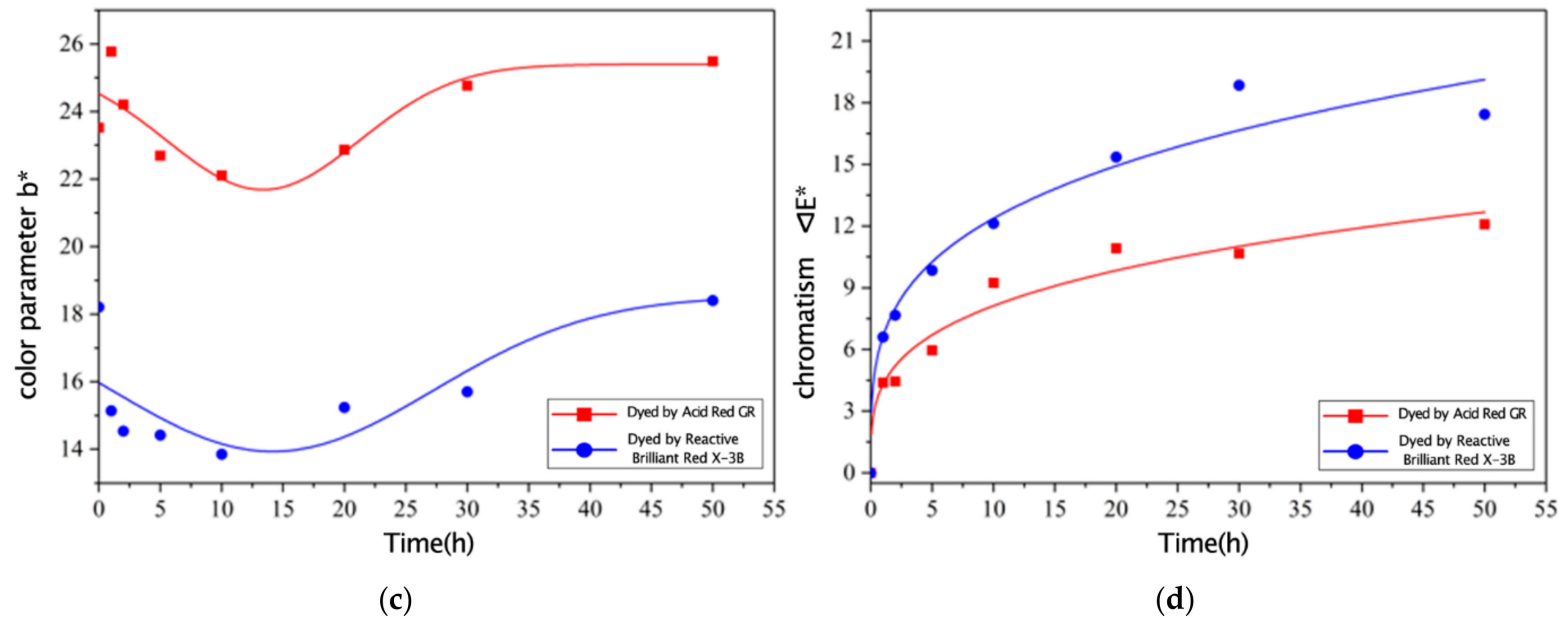
Color comparison of corn straw dyed with the two dyes during light irradiation.

In the accelerated aging process of light irradiation, the brightness index L* curve of the two dyes dyeing corn straw is shown in Figure 4a, the curve of the chromaticity index a*, changing with the illumination time, is shown in Figure 4b, the curve of the chromaticity index b*, changing with the illumination time, is shown in Figure 4c, and the curve of value of chromatism ΔE* is shown in Figure 4d.

In Figure 4a, the measured data showed that the change of the brightness index L* of the two dyed straw was basically the same, and both curves showed an increasing trend. The brightness index L* of Reactive Brilliant Red X-3B-dyed corn straw showed a slower increase than that of corn straw dyed with Acid Red GR, indicating that the brightness change of corn stalk dyed with Reactive Brilliant Red X-3B was slighter after irradiation with ultraviolet light. The curves of dyed straw chromaticity index a* showed a decreasing trend, while the curves of the chromaticity index b* adopted first and then rose, shown in Figure 4b,c, corresponding to the incremental reflection of whiteness, indicating the discoloration of the surface of straw.

It can be seen from Figure 4d that the value of chromatism ΔE* of each group increased with the extension of the irradiation time. The dyed straw underwent significant fading. After 50 h of light irradiation, the value of chromatism ΔE* of the two test pieces exceeded to12NBS, and the maximum was 17.43NBS. The chromophore system of the dyed straw consisted of the dye, lignin, and extractives present in the straw, which combined the chromophore group and the auxiliary color group in many forms and absorbed the visible light [19,20]. In the early stage of light irradiation (the first 30 h), the main reason for the fading of dyed straw is that the dyed material absorbs light and exposes the unsaturated groups in the dye and wood extract, causing photooxidation and photodegradation and bringing irreversible changes and destruction of the dyeing specimen. After 30 h of light irradiation, the chemical structure of the surface of the dyed straw and the active groups in the chromogenic system were basically deteriorated, and other stable groups were formed by the photooxidation and photodegradation. The dyed straw entered a slow deterioration stage, and the color gradually became lighter.

After 50 h of light irradiation, the value of chromatism ΔE* (6.61NBS~17.43NBS) of the straw dyed with Reactive Brilliant Red was significantly larger than that of the straw dyed with Acid Red GR (4.39NBS~12.09NBS). This is because Acid Red GR combined with straw by physical adsorption. The dye molecules adsorbed on the cell wall of the straw were the first to undergo photo-discoloration when light was irradiated. The Acid Red GR is an azo dye with good light resistance [21]. In addition, acid dyes mainly dye lignin in straw components, and photodegradation of lignin is the main cause of wood color fading. The corn straw which was dyed with a reactive dye had the characteristics of high dyeing rate and poor light resistance, maybe because the surface wax of the straw was removed, and the fiber structure of the straw epidermis became weak after pretreatment. The structure of the straw is mainly composed of cellulose and hemicellulose, and the inner core is mainly composed of lignin. After removing the surface wax, the reactive dye could easily bind to the surface fibers [19]. However, the pretreatment process promoted the combination of reactive dyes and surface fibers, and the fiber structure became fragile. With the prolongation of the light time, the fiber structure on the surface of the straw gradually broke, so the light resistance of the straw dyed with the reactive dye was not as good as that dyed with the acid dye. The above parameters are shown in Table 2.

### 3.3. Surface Chemical Structure Changes

**Figure 5:**
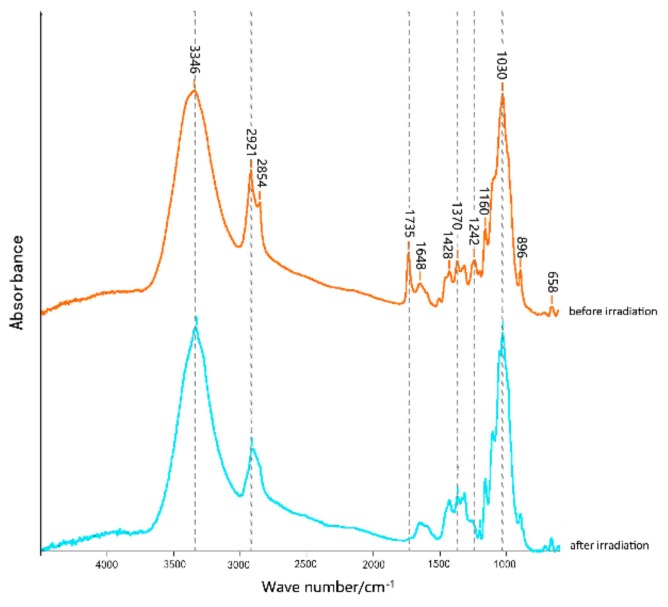
FTIR of corn straw dyed with Acid Red GR under irradiation.

**Figure 6:**
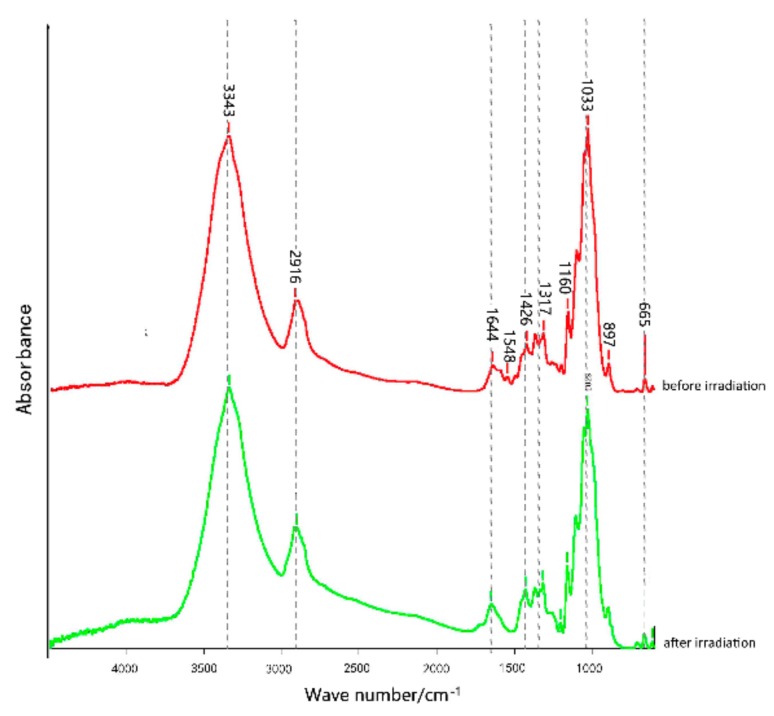
FTIR of corn straw dyed with Reactive Brilliant Red X-3B.

A Fourier-transform infrared spectrometer was used to calculate the infrared spectrum data of corn straw dyed with Acid Red GR and Reactive Brilliant Red X-3B before and after illumination. The results of the analysis are shown in Figure 5 and Figure 6.

The FTIR spectra of the corn straw dyed with Acid Red GR before and after ultraviolet radiation are shown in Figure 5. Considering the peak of 1300 cm^−1^ as a reference, obvious transmittance changes can be observed in the fingerprint region from 800 cm^−1^ to 1800 cm^−1^. The light sensitivities of the carbonyl structure (C=O) and the aromatic ring (C=C) are particularly noticeable. Under irradiation with artificial light, some unsaturated functional structures in lignin and/or dyes were degraded by photooxidation, confirmed by the considerable disappearance of C=O bonds at 1242 cm^−1^ and 1735 cm^−1^. The absorption peaks at 2921 cm^−1^ and 2854 cm^−1^ were significantly reduced, reflecting the C–H stretching vibration in lignin [21,22,23]. These changes indicate that the original chromophoric system of the dyed straw was reduced, inducing an undesirable discoloration. The narrow change of the spectral band at 3346 cm^−1^ reduced the phenolic hydroxyl group –OH, which was gradually broken after 50 h of irradiation. This was probably due to the reaction of the methoxy group on the benzene ring and/or to hydrogen bond cleavage caused by the evaporation of water molecules.

Lignin is an excellent light absorber whose molecular structure can undergo free-radical reactions after irradiation and form new carbonyl and carboxyl chromophoric groups. These molecular structures include carbonyl groups, phenolic hydroxyl, methoxy, and other functional groups [8,15,20]. In this process, lignin degrades through free-radical reactions. Meanwhile, the spectral band of cellulose and hemicellulose at 1426 cm^−1^, 2916 cm^−1^, 1370 cm^−1^, 896 cm^−1^, and 1160 cm^−1^ were almost unaffected by UV irradiation. It can be deduced that Acid Red GR mainly combined with lignin. After UV irradiation, lignin (including aromatic extractives) was reduced and formed new chromophoric groups which caused surface chromatism of the straw.

The FTIR spectra of the corn straw dyed with Reactive Brilliant Red X-3B before and after ultraviolet radiation are shown in Figure 6. Compared with the straw dyed with Acid Red GR, the spectra are similar, especially, the peaks of cellulose and hemicellulose at 1426 cm^−1^, 2916 cm^−1^, 897 cm^−1^, and 1160 cm^−1^ were almost unaffected by UV irradiation. The absorption peak at 1644 cm^−1^ was enhanced, indicating that the hydroxyl group –OH in cellulose could be oxidized to a ketone carbonyl group after irradiation and to an aldehyde, carbonyl group, or carboxyl group by free-radical reactions, and these chromophore groups were unstable under the applied illumination conditions. The narrow change of the spectral band at 3343 cm^−1^ represents the phenolic hydroxyl group (–OH) which was gradually broken after 50 h of irradiation. This was probably due to the reaction of the methoxy group in the benzene ring and/or to hydrogen bond cleavage caused by the evaporation of water molecules.

### 3.4. Surface Microstructure Changes

**Figure 7:**
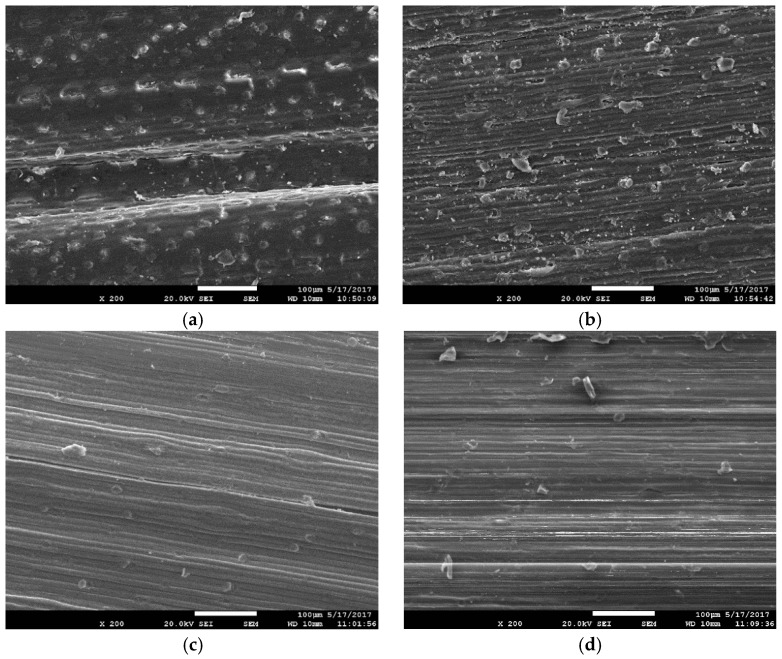
(**a**) Surface of untreated straw; (**b**) Surface of pretreated straw; (**c**) Straw surface dyed with Acid Red GR; (**d**) Straw surface dyed with Reactive Brilliant Red X-3B.

The appearance of untreated, pretreated, and dyed corn straw slices is shown in Figure 7.

**Figure 8:**
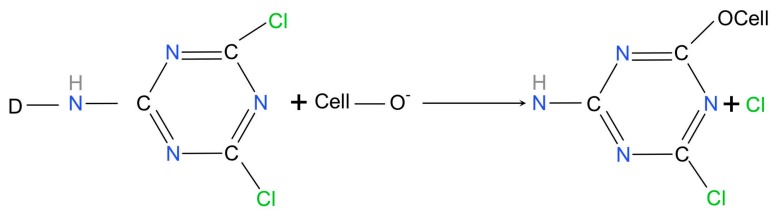
Mechanism of reaction between Brilliant Red X-3B and straw.

It can be seen from Figure 7a that the surface of the corn straw was covered by a waxy layer, whose pore distribution was relatively uniform. After pretreatment, the surface of the straw became rough and many breaks appeared, meanwhile the fiber texture was clear, as shown in Figure 7b. This is because the wax on the surface of the straw was an ester composed of fatty alcohols and higher fatty acids, which was soluble in alkali. In the presence of sodium hydroxide (NaOH), the wax and pectin were dissolved. After the barrier was removed, penetration and diffusion of the dyes were possible. It can be seen from Figure 7c,d that the surface of the straw became smoother, and the texture was clear after dyeing. It can be inferred that there was a physical or organic combination between dye’s molecules and straw microregions. As for Acid Red GR, it exists in aqueous solution as ions (D− or D+), ionic micelles (nDn− or nDn+), and micelles {[(HD)_m_nD]n − or [(DA)_m_n D]n+}. The electrostatic repulsion between the dye SO_3_H-dissociated sulfonic acid group SO_3_^−^ and the negatively charged region in the fiber caused aggregation and blockage of the dye molecules. As for Reactive Brilliant Red X-3B, it can chemically react with cellulose to form ester and ether bonds after the dissociation of the hydroxyl groups in cellulose and forms hydroxyl anions. By combining FTIR and SEM, the penetration or organic binding of Brilliant Red X-3B could be demonstrated, whose mechanism of reaction as shown in Figure 8. In the first step, the ionization of cellulose under alkaline condition and form cellulose anions. In the second step, the cellulose negative ions attack the carbon atoms with the lowest electron cloud distributed around the active group, causing a nucleophilic substitution reaction.

Step 1:
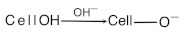
Step 2: Figure 8

### 3.5. Water-Washing Firmness

**Figure 9:**
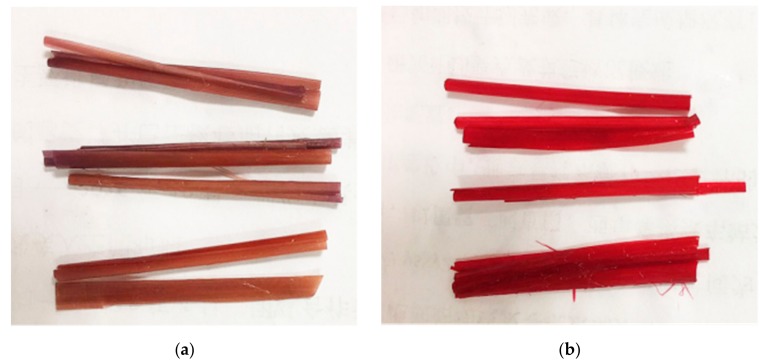
(**a**) Water washing effect of the straw dyed with Acid Red GR (**b**) Water washing effect of the straw dyed with Reactive Brilliant Red X-3B.

After the washing treatment, the surface color of the straw dyed with Acid Red GR became obviously lighter, and the surface color of the straw dyed with Reactive Brilliant Red X-3B remained bright red, as shown in Figure 9.

It can be seen from Table 3 that the chromaticity value ΔE* of the corn straw dyed with Acid Red GR was greatly reduced, and its color was lighter after water washing, while the chromaticity value ΔE* of the corn straw dyed with Reactive Brilliant Red X-3B changed little, and the color did not change obviously at the naked eyes. This is because the dye molecules of Acid Red GR cellulose only physically combined with the lignin of corn straw, so the bonding strength was fragile [19]. When the dyed straw was immersed in hot water, since the concentration of the dye in the water was zero, a large concentration gradient was generated between the inside of the straw and the external environment. With the prolongation of the soaking time, the dye molecules in the straw desorbed and diffused into the water, which produced a great change of the chromaticity value. The combination of Reactive Brilliant Red X-3B and straw was mainly based on covalent bonds, and Reactive Brilliant Red X-3B mainly acted on cellulose, becoming a part of the components of the straw, so Reactive Brilliant Red X-3B did not desorb, and the color changed little.

### 3.6. Utilization of Dyed Corn

**Figure 10:**
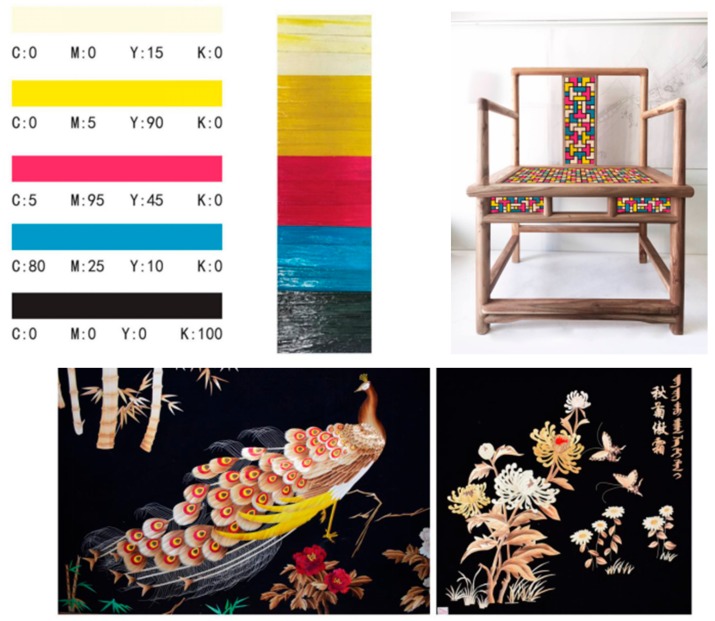
Utilization of dyed straw

Dyed corn straw can be utilized in decorating furniture as pasteup. Corn straw dyed with various reactive dyes is used for decoration elements to improve their color system and aesthetics, as shown in Figure 10.

## 4. Conclusions

1. Corn straw was bleached with 27% hydrogen peroxide (H_2_O_2_) and 0.1% NaOH, which had a good effect of decolorization. The dilute solution of NaOH could significantly improve the permeability of straw and opened the partially blocked pores of the straw, facilitating dyeing.

2. Under the condition of 1 h dyeing time, the dyeing rate of corn straw dyed with Reactive Brilliant Red X-3B was 46%, while the dyeing rate of corn straw dyed with Acid Red GR was 29%, indicating that Reactive Brilliant Red X-3B has a high dyeing rate for corn straw. The most content of cellulose in corn straw is high, indicating that Reactive Brilliant Red X-3B mainly binds cellulose, whereas Acid Red GR mainly binds lignin. 

3. After 50 h of irradiation, the brightness change of corn straw dyed with Reactive Brilliant Red X-3B was small. The values of chromatism ΔE* of the corn straw dyed with Acid Red GR and Reactive Brilliant Red X-3B were 12.09NBS and 17.43NBS, respectively, indicating that the straw dyed with Acid Red GR had a good light resistance.

4. The results of FTIR showed that Acid Red GR mainly combined with lignin, and the lignin (including aromatic extractives) distributed on the surface of the straw was seriously damaged after ultraviolet irradiation, when new chromophoric groups were generated, resulting in surface discoloration of the straw. Reactive Brilliant Red X-3B mainly combined with cellulose and hemicellulose in the corn straw specimens and formed covalent bonds. After the straw was irradiated with ultraviolet radiation for a long time, the fiber inside the straw broke, so that the binding rate between dye and fiber was reduced.

5. SEM showed that when 27% H_2_O_2_ and 0.1% NaOH were used to bleach the corn straw, removing the silica (SiO_2_) covering the surface of the straw, obvious damage on the surface of the straw occurred, which was conducive to the organic combination of dye molecules with the internal components of the straw. After dyeing, the surface of the straw became smoother, and its texture was clear, which proved the infiltration or organic combination of the dye.

6. The analysis of water-washing firmness showed that the value of chromatism ΔE* of corn straw dyed with Reactive Brilliant Red X-3B changed little after soaking at 65 °C for 2 h, and the color did not change obviously at the naked eyes, confirming that the combination of Reactive Brilliant Red X-3B with straw was based mainly on covalent bonds, while Reactive Brilliant Red X-3B mainly acted on cellulose and became a component of the straw.

## 5. Patents

Patent of invention: A method of pretreatment and dyeing of corn straw, grant number ZL201710873966.X.

## Figures and Tables

**Table 1:** Dyeing rate of Acid Red GR and Reactive Brilliant Red X-3B. A_0_: absorbance before dyeing, A_1_: absorbance after dyeing.

**Acid Red GR**					
	**Test 1**	**Test 2**	**Test 3**	**AVG**	Dyeing Rate/%
A0	0.716	0.715	0.721	0.717	29
A1	0.497	0.512	0.516	0.508	
**Reactive Brilliant Red X-3B**					
	**Test 1**	**Test 2**	**Test 3**	**AVG**	Dyeing Rate/%
A0	0.789	0.785	0.781	0.785	46
A1	0.420	0.432	0.424	0.425	

**Table 2:** Photochromic parameters of Acid Red GR and Reactive Brilliant Red X-3B.

**Acid Red GR**							
**Time(h)**	L*	a*	b*	△L*	△a*	△b*	ΔE*
0	37.92	42.28	23.52	0	0	0	0
1	41.57	41.34	25.77	3.65	−0.94	2.25	4.39
2	40.7	38.88	24.21	2.78	−3.4	0.69	4.45
5	40.85	37.16	22.69	2.93	−5.12	−0.83	5.96
10	42.7	34.51	22.11	4.78	−7.77	−1.41	9.24
20	43.18	32.73	22.87	5.26	−9.55	−0.65	10.92
30	43	32.97	24.77	5.08	−9.31	1.25	10.67
50	44.68	32.45	25.49	6.76	−9.83	1.97	12.09
**Reactive Brilliant Red X-3B**							
**Time(h)**	L*	a*	b*	△L*	△a*	△b*	ΔE*
0	37.73	51.74	18.21	0	0	0	0
1	37.62	45.89	15.14	−0.11	−5.85	−3.07	6.61
2	37.8	45.02	14.54	0.07	−6.72	−3.67	7.66
5	38.48	42.69	14.42	0.75	−9.05	−3.79	9.84
10	39.11	40.51	13.85	1.38	−11.23	−4.36	12.13
20	40.56	36.95	15.24	2.83	−14.79	−2.97	15.35
30	41.36	33.42	15.7	3.63	−18.32	−2.51	18.84
50	41.7	34.77	18.4	3.97	−16.97	0.19	17.43

**Table 3:** Chromaticity values of straw before and after washing.

Dyeing Materials	ΔE* before Washing	ΔE* after Washing	Change of ΔE*
Acid Red GR	4.39	1.29	−3.1
Reactive Brilliant Red X-3B	6.61	6.24	−0.37
